# Acoustic and Perceptual Profiles of Swallowing Sounds in Preterm Neonates: A Cross-Sectional Study Cohort

**DOI:** 10.1007/s00455-025-10807-5

**Published:** 2025-02-11

**Authors:** Thuy T. Frakking, Seiji Humphries, Anne B. Chang, Belinda Schwerin, Majorie M. Palmer, Michael David, Annelise Kyriakou, Stephen So, SUPERB collaborative, Manuel Bautista, Alicia Blake, Julie Dunsmuir, Timothy Hong, Kelly Weir, Christopher Carty, Paul Colditz, Melissa Lai

**Affiliations:** 1 Research Development Unit, Caboolture Hospital, Metro North Health, McKean St, Caboolture, QLD 4510 Australia; 2 Child Health Research Centre, School of Medicine, The University of Queensland, South Brisbane, QLD 4101 Australia; 3 Speech Pathology Department, Gold Coast University Hospital, Gold Coast Health, 1 Hospital Boulevard, Southport, QLD 4215 Australia; 4 School of Health Sciences & Social Work, Griffith University, 1 Parklands Drive, Southport, Gold Coast, QLD 4222 Australia; 5 Department of Respiratory Medicine, Queensland Children’s Hospital, 501 Stanley St, South Brisbane, QLD 4101 Australia; 6 Child Health Division, Menzies School of Health Research, Charles Darwin University, PO Box 41096, Casuarina, NT 0811 Australia; 7 Australian Centre for Health Services Innovation, Queensland University of Technology, Level 7, 62 Graham St, South Brisbane, QLD 4101 Australia; 8 School of Engineering and Built Environment, Griffith University, Parklands Dr, Southport, QLD 4215 Australia; 9 NOMAS International, San Juan Bautista, CA USA; 10 The Daffodil Centre, The University of Sydney, a joint venture With Cancer Council, Sydney, Australia; 11 School of Medicine and Dentistry, Griffith University, Gold Coast, QLD 4222 Australia; 12 Peninsula Plus, Speech Pathology Team, Frankston, VIC 3199 Australia

**Keywords:** Cervical auscultation, Deglutition disorders, Oropharyngeal aspiration, Deglutition, Swallow sounds, Preterm, Neonate

## Abstract

Cervical auscultation, commonly used by speech-language pathologists in some countries as an adjuvant to the clinical feeding evaluation, requires data on acoustic and perceptual profiles of swallowing sounds. Whilst these exists in adults and children, none currently exist for preterm neonates. Our study aims to establish the acoustic and perceptual parameters of swallowing sounds in preterm neonates. Swallowing sounds were recorded on a digital microphone during oral feeding observations. Acoustic parameters of duration, peak frequency, peak power and peak intensity were determined. Perceptual parameters heard pre, during and post-swallows were rated as ‘present’, ‘absent’, or ‘cannot be determined’. Eighty preterm neonates (43 males; mean age = 33.4 weeks [SD 2.6]) from three Australian special care nurseries demonstrated mean swallow durations of < 1 s. The peak amplitude correlated with the number of medical co-morbidities (*r* = 0.24; 95%CI 0.03–0.45). Most preterm neonates have coordinated swallows that are loud, quick and completed in < 1 s. The perceptual parameters of a bolus transit sound was consistently present in all preterm neonates. One in five pre-term neonates have an uncoordinated swallow where wheeze, stridor or wet breath sounds were present post-swallow. Our study provides clinicians with acoustic and perceptual parameters to guide use of cervical auscultation in special care nurseries. Future studies should consider simultaneous instrumental assessment to ensure validity when using cervical auscultation to support diagnostic decision-making on swallowing coordination.

**Supplementary Information:**

The online version contains supplementary material available at 10.1007/s00455-025-10807-5.

## Introduction

About 10% of preterm neonates have feeding difficulties, with higher rates seen in preterm neonates with very low birth weight or chronic neonatal lung disease [1–3]. Nearly one in two preterm neonates with feeding difficulties will show signs of oropharyngeal aspiration (abbreviated to aspiration), with a majority demonstrating no overt clinical signs of aspiration (e.g. cough) [3, 4]. In addition to the absence of overt clinical signs, the underlying pathophysiology for preterm neonates’ feeding difficulties is not fully understood although it is known that their neurosensory and neuromotor systems are still maturing and related with their developmental gestational age [5–7]. Thus, healthcare professionals (e.g. speech-language pathologists, occupational therapists) often use additional validated assessments (e.g. Early Feeding Skills Assessment Tool, Neonatal Oral-Motor Assessment Scale) [8–11] in conjunction with the clinical feeding examination to increase their objectivity in the assessment of feeding difficulties in preterm neonates.

A videofluoroscopic swallow study (VFSS) is considered the gold-standard assessment for the assessment of aspiration in preterm neonates because it captures all phases of the swallow, including the direct visualization of aspiration events [12]. However, using VFSS to assess preterm neonates’ feeding has many limitations including: a) concerns of the cumulative exposure to ionizing radiation [13–15]; b) their oral feeding performances can vary thus making interpretation of a single point in time difficult [8]; c) VFSS cannot replicate breastfeeding without exposing the mother’s breast tissue to radiation as well [16]; and d) infant formula or breastmilk mixed with barium impregnated fluids may differ in texture and viscosity than what is offered outside of VFSS [17, 18]. While the fiberoptic endoscopic evaluation of swallowing (FEES) is an alternative to the VFSS [19], access to it is limited as it requires specialized equipment and high-level expert health professionals conducting and interpreting it. Given the limitations of VFSS and FEES, alternative assessments that are accessible, repeatable and has low costs would help in the assessment of feeding issues among the preterm neonatal population.

Cervical auscultation (CA) is a non-invasive, low cost, highly accessible and repeatable assessment technique commonly used as an adjuvant to the clinical feeding evaluation in the United Kingdom, Ireland and Australia [20, 21]. CA involves using a stethoscope, digital accelerometer or digital microphone to capture swallow and breath sounds generated during the oral preparatory, oral and pharyngeal phases of swallowing [22, 23]. Diagnostic test accuracy studies have demonstrated that digital CA has high sensitivity 0.85 (95%CI 0.62–0.97), high negative predictor value 0.92 (95%CI 0.78–0.98), very good inter-rater (0.81; 95%CI 0.79–0.84) and good to very good intra-rater (kappa range 0.72–0.98) reliability in detecting aspirating swallows, compared with VFSS in term neonates and children with paediatric feeding disorders at a pediatric tertiary hospital [24, 25]. The accurate use of cervical auscultation in clinical practice typically requires structured training to reliably interpret swallow sounds and swallow-respiratory coordination [26]. This may include theoretical and practical training on swallow and respiratory sounds recorded from children and adults with and without dysphagia. Specifically, sound features of the swallow [27] and post-swallow breath [22, 28] sounds can be reliably differentiated between aspirating and non-aspirating swallows in children with dysphagia, including children with Trisomy 21. These sound features require knowledge on acoustic and perceptual swallowing sound profiles. Recent further improvements to the objectivity of CA in adults and children include using classifiers that can differentiate between impaired and non-impaired swallows [27, 29–34]; and CA sound features correlates with swallow kinematic events such as hyoid bone displacement [35–37], laryngeal closure [38] and upper oesophageal sphincter opening. [39]

Despite the increasing evidence on the clinical utility of digital CA to detect aspiration, data on CA in preterm neonates are limited. Seminal work by Reynolds and colleagues [40, 41] demonstrated that the variance index (based on swallowing sound waveforms) increased in signal consistency as preterm neonates reached term age; and that there were differences in swallowing sound waveforms between preterm neonates with and without chronic neonatal lung disease. However, both studies were limited by their small sample sizes (n = 10 and 17, respectively), use of inferior audio recording technology compared to technologies presently available and also lacked objective assessments (e.g. gold standard VFSS) to quantify normal versus aspirating swallows in their preterm cohorts. Recently, Yu-Lin et al [42] used CA in conjunction with a sucking pressure device to develop a suck-swallow-breathing detection algorithm and reported a sensitivity of 91% for the detection of swallows in term neonates. Yu-Lin et al.’s [42] work applying machine learning to preterm swallows is promising but direct translation to clinical practice is difficult because the equipment and developed algorithm are not yet widely available for clinical use. Thus although clinicians are already using CA and swallowing sound profiles to guide the interpretation of swallowing sounds heard, its use among preterm neonates has several critical gaps including the known differences in the oromotor feeding skills of preterm and term neonates in relation to sucking rhythmicity, swallowing rates, length and efficiency of suck bursts, and swallowing physiology [43–46]. Further, there are many other factors in preterm neonates that potentially influence swallowing sound parameters (e.g. amplitude and frequency), such as their smaller aerodigestive tract size, increased respiratory rates, reduced muscle tone and fatty tissue in the oropharynx, compared to older infants [47]. Normative acoustic (e.g. frequency, duration, amplitude) and perceptual clinical (e.g. clear breath sounds, bolus transit sound, wet breathing) swallowing sound parameters have been reported in newborns [48], infants [49, 50], children 3–11 years [48, 51], and adults [52–56]. There is a need to establish similaracoustic and perceptual clinical swallowing sound profiles in preterm neonates to facilitate CA use within the preterm neonatal population because no such reference data currently exists.

## Method

### Study Design

This multisite cross-sectional study across 3 Australian special care nurseries with level 6 (Royal Brisbane & Women’s Hospital and Gold Coast University Hospital) and level 4 (Caboolture Hospital) clinical services capability [57] received ethical approvals across Queensland Health (HREC/2021/QRBW/76999) and Universities (2022/HE000128 and GU Ref No: 2022/129). Parents or guardians provided written informed consent.

### Participants

Inclusion criteria: gestational age ≤ 36^+6^ weeks, tolerating bolus feeding, evidence of infant-driven feeding scale scores of 1 or 2 at oral feeding times (e.g. rooting or taking to pacifier, adequate tone and drowsy or alert/fussy once handled) [58], and a normal or disorganized categorization on the NOMAS [59] [independently rated by external speech pathologist (MMP)], evidenced by no dysfunctional oral-motor ratings (e.g. asymmetry/lateral jaw or tongue deviation, excessively wide or minimal excursions, absence of movement, lack of rate change, flaccid/retracted tongue). The Early Feeding Skills Assessment Tool [8, 60] was used for preterm neonates who did not provide a sustained 2-min oral feeding performance for valid NOMAS [59] rating. Preterm neonates were excluded if they had any structural (e.g. cleft lip or palate) or chromosomal abnormality that affected feeding/swallowing ability. Nasogastric tube feeding was not an exclusion criteria because the presence of nasogastric tube feeding is commonly seen in preterm neonates who are on a combination of oral and tube feeds while establishing full oral feeding. We hypothesize that the presence of the nasogastric tube does not affect the swallowing sounds collected based on previously published literature that found no significant difference in penetration/aspiration adults with or without nasogastric tube [61]. More recently, the application of machine learning based on cervical auscultation recorded sounds in adult patients without nasogastric tube provides proof of concept that sounds from patients without nasogastric tube could successfully generalise to predict swallow kinematic events in adults with nasogastric tube. [62]

### Procedure

Audio-visual recordings of feeding observations of preterm neonates breast or bottle feeding were collected. Two-minute segments from the feeding observations were extracted for use in this study using an acoustic editing software package (Adobe Audition CS6 v5, Adobe Systems, USA). All preterm neonates were fed in standard feeding positions (e.g. elevated side lying, semi-reclined position) by their caregiver or treating nurse.

### Equipment

An omnidirectional condenser microphone (C417, AKG Acoustics, Vienna, Austria) (sensitivity at 1 kHz of 10 mV/Pa, impedance 200Ω, frequency range 20 to 20,000 Hz) [63] was connected to a digital video recorder (Model XA40, Cannon Incorporation, Tokyo, Japan) for the simultaneously recording of swallowing sounds and visual data. Using microfoam taping, the microphone (inserted into a fitted O-ring) was placed onto the neonate’s neck immediately lateral to the midline position. Sound quality throughout the feeding observation was monitored with headphones (Model ATH-M50x, Audio-Technica, Taiwan) directly connected to the digital video recorder by researcher (TTF). The position of the microphone was adjusted as required, when the ambient room noise exceeded swallowing sound quality. This occurred when there was a reduction in contact between the microphone and skin surface.

### Identification of Swallow Sounds from Audiofiles

Using the acoustic editing software package described above, the raw acoustic signal was displayed as a waveform plot (y-axis (amplitude, decibels, dB) versus x-axis (time, seconds, s) (44,100 Hz sampling rate) simultaneously with the visual recording of the neonate breast or bottle feeding and a spectrogram (y-axis: frequency, Hz; x-axis: time, s). The waveform plot was magnified so that a 1-s section of the audio waveform filled the majority of the program’s viewing window. The start and end of each individual swallow was characterized simultaneously with the audio feedback of the swallowing sound (i.e. presence of a fluid flushing sound immediately after the cessation of inspiratory/expiratory respiratory sounds), visualization of the swallow from the visual recording (i.e. wide jaw excursion) and visualization of the spectrogram (i.e. areas of intensity).

The accurate use of cervical auscultation requires structured training to reliably interpret swallow sounds and swallow-respiratory coordination [26]. The timepoints for swallow sounds were identified by two researchers who have received cervical auscultation training to reliably interpret swallow sounds: TTF, a pediatric speech pathologist with postgraduate doctoral skills in cervical auscultation, and SH, a PhD-qualified health researcher. Initially, TTF and SH completed the initial identification of swallow sound timepoints for five preterm neonates together. Following this, SH completed the identification of swallow sound timepoints (n = 3414) for the remaining cohort for review by TTF. Any disagreements were resolved by discussion and achievement of a consensus value between the two raters.

### Data Collection

#### Acoustic Parameters

Segmented swallow interval times (start and end points of the swallow) were extracted and analyzed for duration, peak frequency, peak amplitude and peak power using a Python program. Duration (in seconds) was defined as the total time elapsed from the beginning to the end of the swallowing sound. Peak frequency (Hertz, Hz) refers the highest frequency contained in the time segment of maximum energy. The signal was first filtered using a high pass fourth order Butterworth filter with a cutoff frequency of 400 Hz to remove large energy components in the low frequency range (0 – 400 Hz). The filtered signal was then divided into short 20 ms frames with a 50% overlap. The power spectral density (PSD) was then estimated for each frame using the Welch periodogram method [64] (using 10 sub-frames with 50% overlap) and then normalized. The Welch method was chosen for its ability to compute low variance estimates of the PSD. [65]

To determine peak frequency, a cumulative distribution function (CDF) of the PSD was constructed going from low to high frequency. This CDF was then used to determine the frequency range that captured or represented 95% of the total signal power in the frequency domain. This method relies on a more intuitive criteria for determining the peak frequency, as opposed to the arbitrary noise flooring that is typically used to enhance visual interpretation in spectrogram analysis.

Peak amplitude (decibels, dB) was defined as the maximum signal value that was found in the swallow. To calculate the peak power, the total signal energy in each 20 ms frame was first computed and divided by the duration, so as to provide the signal power for that frame. The highest signal power was then determined to be the peak power. Any clipped samples were removed (and signal duration adjusted accordingly) so as not to bias the power measure.

#### Perceptual Clinical Parameters

The perceptual clinical parameters (Supplemental Table B, adapted from previously published normative data in children) [49] for breath/swallow sounds heard throughout the swallow phases in the 2-min audio-visual recordings were rated independently by two speech-language pathologists (TTF & AK), with 18 and 4 years’ experience in pediatric feeding disorders. Both TTF and AK have attended formal CA workshops, currently use CA in clinical practice and conduct CA research. Across the entire series of swallows identified in the 2 min audio file, sounds pre-, during and post-swallow sounds ratings were nominal (yes/no/cannot be determined) and were marked as present if the parameter was demonstrated on at least one occasion. Additionally, sounds marked as present for during swallow sounds were also rated for consistency (yes/no). A consistent swallow sound was defined as present in > 80% of all possible swallows in the audio file. A coordinated swallow sound was defined as the presence a bolus transit sound and normal breathing post-swallow or no change to breath sounds pre- and post-swallow. An uncoordinated swallow sound was defined as the presence of a bolus transit sound and an absence of normal breathing post-swallow and/or glottal release sound plus one or more of the following post-swallow: wet breathing, rattly chest, cough, wheeze, crackles, throat clearing, stridor [27]. The definitions of coordinated and uncoordinated swallows were based on previous studies on cervical auscultation where VFSS was used to determine the absence/presence of aspiration [22, 28, 49]. Disagreements with ratings were resolved by discussion and a consensus rating agreed between TTF & AK. Consensus was used for finalization of ratings in our study as it is a widely used and accepted resolution mechanism in the research literature. [66]

### Statistical Analysis

We planned for a sample size of 80 preterm neonates with 80% power (two-sided α = 0.05, difference ≥ 0.31 between acoustic parameter variables) [67]. Data analysis was completed with Stata (v12.0, StataCorp, Texas, USA). Normality of data was checked using the Shapiro–Wilk normality test [68]. Means (SD, standard deviations) were computed for the acoustic parameters of peak frequency, duration, peak amplitude and peak power. Pearson’s correlation coefficient was used for correlations between continuous variables. A Spearman’s correlation analysis between age (gestational, birth), number of medical conditions and acoustic parameters of peak frequency, duration, peak amplitude and peak power was conducted. The consensus ratings for perceptual clinical parameters pre-, during and post-swallows present for each preterm neonate were used to calculate percentages. In addition, classifications of swallowing sounds were compared using percentage agreement and Cohen's Kappa coefficient [69, 70]. Cohen's Kappa coefficients were calculated for each stage of swallowing and perceptual sound parameter. Since percentage agreement does not account for chance agreement and Cohen's Kappa is prone to underestimation due to skewed prevalence or systematic bias in rater classifications, Prevalence-Adjusted and Bias-Adjusted Kappa (PABAK) coefficients were also calculated to determine agreement [71]. Interpretation of reliability results for both coefficients was based on the following criterion [72]: 0.80 – 1.00 Excellent; 0.61 – 0.80 Substantial; 0.41 – 0.60 Moderate; 0.21 – 0.40 Fair; < 0.20 Slight to Poor.

## Results

A total of 80 preterm neonates were recruited (n = 43 male; mean birth gestation 33.4 SD 2.6 weeks) between October 2021 to July 2022. The most common medical conditions were jaundice, hyaline membrane disease (HMD) and intrauterine growth restriction (IUGR). Over half the cohort required respiratory support during their admission, with no infants on any respiratory support at the time of their feeding observation. 52% of preterm neonates were on a combination of oral and tube feeding at the time of their feeding observation (Table 1).

**Table 1:** Baseline characteristics of our cohort

Characteristics	Total (N = 80)
Birth gestational age (weeks)	
Mean (SD)	33.4 (2.6)
Median (IQR)	34 (32–35)
Range	25 – 36
Birth weight (grams)	
Mean (SD)	2109.6 (560.3)
Median (IQR)	2140 (1750–2470)
Range	568 – 3545
Chronological gestational age at feeding observation (weeks)	
Mean (SD)	37 (1.6)
Range	34 – 43
Sex	
Male	43 (53.8%)
Female	37(46.2%)
Multiple birth	32 (40.0%)
Feeding status at feeding observation (n = 77)	
Full oral	25 (32.5%)
Combination (feeding tube and oral feeds)	52 (67.5%)
Days of tube feeding (n = 77)	
Mean (SD)	26.4 (25.3)
Median (IQR)	18.5 (13–27)
Range	1 – 133
Respiratory support required during admission	62 (77.5)
Hours of respiratory support (n = 55)	
Mean (SD)	93.5 (191.8)
Median (IQR)	25 (10–50.9)
Range	0.1 – 921.7
Common Medical conditions	
Hyaline Membrane Disease (HMD)	35 (43.8%)
Intrauterine growth restriction (IUGR)	34 (42.5%)
Hypoglycaemia	31 (38.8%)
Presumed sepsis	3 (3.9%)
Management of monochrionic twin (MCDA)	3 (3.9%)
Apnea	11 (13.8%)
Jaundice	51 (63.8%)
Chronic neonatal lung disease (CNLD)	5 (6.3%)
Transient tachypnoea of the newborn (TTN)	9 (11.3%)
Persistent pulmonary hypertension	2 (2.5%)
Retinopathy of prematurity (ROP)	7 (8.8%)
Non-specific respiratory disease	3 (3.9%)

Data are mean (SD), n (%)

*SD* Standard deviation, *IQR* Interquartile range

### Acoustic Parameters

The duration of all swallows was mean = 0.85 s (SD0.3). Peak amplitude (mean = 89.7 dB SD1.1) and peak power (mean = 80.8 dB SD4.8) were similar (Table 2).

**Table 2:** Swallowing acoustic parameters for preterm neonates on thin fluids

	Duration (s) Mean ± SD	Peak frequency (Hz) Mean ± SD	Peak amplitude (dB) Mean ± SD	Peak power (dB) Mean ± SD
Thin fluids (N = 80)	0.85 ± 0.3	1678.3 ± 413.5	89.67 ± 1.1	80.83 ± 4.8

*SD* Standard deviation

### Relationship Between Acoustic Parameters, Gestational Age and Number Of Medical Conditions

The peak amplitude (intensity) of thin fluid swallowing sounds significantly positively correlated with the number of medical conditions (r = 0.24; 95%CI 0.03,0.45). There was no correlation between swallowing acoustic parameters and birth or chronological gestational age at time of feeding observation.

### Perceptual Clinical Parameters

#### Inter-rater Reliability

There was moderate agreement for pre-swallow parameters, fair agreement for during swallowing parameters and moderate to substantial agreement for swallowing coordination and post-swallowing sound parameters between raters (Supplementary Table A).

#### Respiratory Sounds

Most of the cohort had normal breathing pre-swallow (n = 62/79, 78.5%). Respiratory sounds were present pre- and post-swallow in this cohort with the most commonly occurring: stridor, wheeze or wet breathing. Post-swallow cough was observed in 5 of 80 preterm neonates (6.3%), Table 3.

**Table 3:** Data on the number of participants with presence of perceptual pre-, during and post-swallow sound parameters on thin fluid consistency

	Thin fluids (N = 80)
	n (%)
Pre-swallow breath sounds	
Normal breathing	63* (78.5)
Wet breathing	14 (17.5)
Rattly chest	0 (0)
Grunting	12 (15)
Crackles	0 (0)
Stridor	26 (32.5)
Wheeze	16 (20)
Throat clearing	0 (0)
Coughing	0 (0)
Swallow sounds	
Crisp “clear”	76 (95)
Quick	80 (100)
Loud	71 (88.8)
Initial discrete sound (IDS)	79 (98.8)
Bolus transit sound (BTS)	80 (100)
Final discrete sound (FDS)	79 (98.8)
Glottal release sound (GRS)	80 (100)
Co-ordinated	65 (81.3)
Unco-ordinated	15 (18.8)
Post-swallow breath sounds	
Normal breathing	45 (56.3)
Wet breathing	16 (20)
Rattly chest	0 (0)
Grunting	7 (8.8)
Crackles	0 (0)
Stridor	33 (41.3)
Wheeze	24 (30)
Throat clearing	5 (6.3)
Coughing	1 (1.3)

*n = 79 due to one consensus rating of cannot be determined

#### Swallowing Sounds

The bolus transit sound (BTS) and glottal release sound (GRS) was present on at least one swallow in all participants. The BTS, along with the quick and loud descriptors were consistently heard. A majority of preterm neonates were rated with a coordinated swallow (65/80, 81.3%), with nearly 20% rated with an uncoordinated swallow during the feeding observation (Table 3).

## Discussion

Our study involving 3414 swallowing sounds in 80 preterm neonates recruited from 3 special-care nurseries provides the largest collection of acoustic and perceptual swallowing sound profiles in preterm neonates. The acoustic parameters for swallowing sounds in preterm neonates showed that all swallows were quick and took ~ 1 s and that our new proposed peak power for describing sound intensity was comparable to peak amplitude values. Specifically, the peak amplitude (intensity) of swallowing sounds significantly positively correlated with the number of medical conditions in our preterm cohort. For perceptual parameters, all preterm neonates demonstrated the presence of a bolus transit sound (BTS) and a glottal release sound (GRS). While most of the cohort were rated with a coordinated swallow, a substantial proportion (20%) rated with an uncoordinated swallow. Further, abnormal respiratory sounds of stridor (41%), wheeze (30%), wet breathing (20%) and coughing (6.3%) post-swallows were observed. Only half of the cohort were classified as having normal breathing post-swallow.

Our findings of < 1 s mean swallow duration in preterm neonates is consistent with previous data for swallowing sounds on thin fluids in children [49–51, 73]. However, the peak frequency (i.e. pitch) in preterm neonates (e.g. 1650 Hz) from this study was lower than previously reported values in children 4 to 36 months. [49] This could be explained by instrumental artifact between studies. Alternatively, the difference in peak frequency could also be explained by the ongoing maturation of oral feeding skills (which includes swallowing coordination) in preterm neonates compared to established oral feeding skills in children. The development of oral feeding skills in preterm neonates relies on the complex integration of multiple subsystems that include respiratory regulation, oral-motor function, swallowing coordination, engagement and physiological stability [8]. Lau and colleagues [47] found that suction amplitude and bolus size increased with time in preterm neonates. Thus, it is plausible that swallowing sounds in children are based on larger bolus sizes associated with increased efficiency, maturation and stability of oral feeding skills when compared to preterm neonates. Youmans and colleagues [54] previously reported that larger bolus sizes were correlated with higher peak frequency sounds in healthy adults. Indeed, previous research in healthy adults demonstrated that larger bolus sizes (e.g. saliva versus water swallows) generated higher velocity [74]. It is anticipated that the velocity of the bolus size also influences the frequency of swallow sounds in preterm neonates. Future studies could explore how different bolus sizes influence swallowing sound acoustics in preterm neonates.

In this work, we proposed the use of peak power rather than the peak amplitude that was used in previous pediatric and adult studies [49, 53]. The justification for this is that signal power or energy over a short time duration correlates better with the loudness of sound than the instantaneous amplitude value. Also, because the signal power is computed as the time-based average of the squared signal amplitude over a short time duration, it tends to be more resistant to spurious impulse-like peaks that could be introduced by external disturbances. Our method of determining the peak frequency relies on a more consistent and objective measure than that cited in previous studies. The common method was based on determining in the spectrogram the frequency at where the energy of the signal ‘ends’. However, most general spectrogram methods remove spectral information whose magnitude or power is below an arbitrary threshold or ‘noise floor’, which allows the peak frequency to be defined. However, the actual noise floor can be different, depending on the recording environment and this variability results in a mismatch with the default threshold that is coded in the spectrogram method. Rather than relying on a fixed and arbitrary threshold that is based on what the noise floor might be, we have developed a more data-driven method for determining the peak frequency that we believe is more objective. Furthermore, we have chosen to use the Welch periodogram method [64] to give lower variance estimates of the power spectral density.

In this study, we found that the number of medical co-morbidities was positively correlated to peak amplitude (i.e. loudness). Indeed, increased multiple co-morbidities in neonates has been associated with reduced full oral feeding success [75]. Neonates with multiple co-morbidities are likely to undergo more procedures (e.g. bronchoscopy, prolonged mechanical ventilation, multiple intubations) [76] and have persistent feeding difficulties after surgery [77]. Medical procedures can impact on the maturation of their self-regulation development, including hypersensitivity to stimulation. Hypersensitivity to the presentation of a teat and subsequent thin fluid flow could result in variable or larger extraction of bolus sizes for swallowing, impacting on oral feeding efficiency. Medoff-Cooper and colleagues [78] showed that oral feeding efficiency in preterm neonates improved with alert states during feeding. We hypothesize that preterm neonates with less medical co-morbidities demonstrate oral feeding readiness cues more consistently and are more efficient with their oral feeding through the extraction of consistent smaller bolus sizes. In comparison, preterm neonates with increased multiple co-morbidities have less consistency in their alert states for oral feeding, resulting in less efficient oral feeding of variable bolus sizes.

Previous studies on cervical auscultation have documented the presence of a BTS or main swallowing sound in preterm neonates [40] and children [49, 51]. Our study confirms that the BTS is also present in a larger cohort of preterm neonates. This is important in the translation of cervical auscultation use in the newborn care setting as the BTS can also be used as a perceptual marker for when nutritive swallows are occurring. Nearly all preterm neonates in our study had the sequence of initial discrete sound-bolus transit sound-final discrete sound-glottal release sound (IDS-BTS-FDS-GRS) present during their feeding observation. There are no comparable studies in preterm neonates, except those described in children [49, 51]. Almeida et al [51] recorded the swallowing sounds of children aged 3 to 11 years (n = 118) on a combination of puree and thin fluids. It is likely that the combination of puree and thin fluids may have reduced the sensitivity of detecting the full sequence of IDS-BTS-FDS-GRS because swallow sounds on puree have less intensity than thin fluids in adults [54] and children [49]. Another reason for a higher detection of IDS-BTS-FDS-GRS may be related to the reduced aerodigestive tract size, muscle tone and fatty tissue in the oropharynx of preterm neonates. We hypothesize that these factors reduce the distance between the thin fluid and microphone; thereby, creating a louder, more audible swallow sound to rate its individual features better.

The presence of ‘wet breathing’ post-swallows has been previously described in infants with down syndrome (0 to 18 months) [28], healthy infants (mean 9 months) [50] and children (mean 17.1 months) [49]. Our study cohort had a higher rate of wet breathing post-swallows and the presence of stridor and wheeze – respiratory sounds that are not typically found in healthy children. Preterm neonates have a compressed oropharyngeal anatomy, compared to longer elongated oropharyngeal anatomy typically seen in children [79]. We hypothesize that the compressed nature of these structures along with elevated sidelying or semi-reclined feeding positions for preterm neonates likely contributes to increased instances of nasal regurgitation of milk during bottle or breastfeeding. This may account for the higher rates of ‘wet breathing’ observations found in our study cohort. The presence of stridor and wheeze could be explained by the characteristics of our preterm cohort, which included conditions such hyaline membrane disease (HMD), intrauterine growth restriction (IUGR) and chronic neonatal lung disease (CNLD). IUGR, significant respiratory disease and CNLD are known risk factors associated with wheeze in preterm neonates [80, 81]. This is supported by a recent study by Zhou and colleagues [82] which used acoustic analyses and showed that the breath sound acoustic profiles differed between preterm and term neonates. It is possible that the presence of stridor in our preterm cohort was exacerbated during oral feeding and/or caused by undiagnosed laryngomalacia. Leonard and Reilly [83] explain that laryngomalacia is the most common cause of stridor in newborns and that occasional intermittent stridor and difficulty with oral feeding is common in preterm neonates. Compared to term neonates, preterm neonates have a higher incidence of tracheomalacia, bronchomalacia, subglottic stenosis and laryngeal edema [77]. Similarly, wheeze is common and more likely present in preterm neonates compared to term neonates [84–86]. Preterm wheeze can be explained by either: a) lung injury from mechanical ventilation, hypoxic and hyperoxic stress; and infection and inflammation, or b) disruptions to developmental changes from early exposures that affect lung growth [87, 88]. Thus, when applying cervical auscultation in the newborn care setting, clinicians can expect to detect increased instances of wet breathing, wheeze or stridor post-swallows during their feeding observations.

The absence of FEES and/or VFSS to objectively confer the presence or absence of coordinated versus uncoordinated swallows was a limitation of this study. Without VFSS or FEES, there was no objective physiologic evidence of swallowing events. We acknowledge that it would be difficult to obtain ethical approval to complete VFSS on healthy preterm neonates. Nevertheless, the definitions used for coordinated and uncoordinated swallows in our study were based on previous studies on cervical auscultation where VFSS was used to determine the absence/presence of aspiration [22, 28]. Additionally, the subjective identification of swallow sound timepoints were based on previous studies in adults [56] and children [27] that correlated cervical auscultation recorded sounds with VFSS. Future studies in preterm neonates could consider using FEES concurrently with cervical auscultation recorded sounds to improve the validity of this technique. Finally, whilst respiratory rates or direction were not assessed in this study, future studies could consider incorporating this information with cervical auscultation to support the presence/absence of coordination given its central role in neonatal swallow-breath interactions [89, 90].

## Conclusion & Future Directions

A majority of preterm neonates have coordinated swallows that are loud, quick and completed in < 1 s. The perceptual swallow sound parameters of a BTS and GRS were present in all preterm neonates. One in five preterm neonates have an uncoordinated swallow where wheeze, stridor and wet breath sounds can be present post-swallow. The acoustic and perceptual swallowing sound profiles provides clinicians with reference values when using cervical auscultation for pre-term neonates typically seen in the special care nursery setting. This study lays the foundation for future comparison to abnormal swallowing sounds in preterm neonates.

## Supplementary Information

Below is the link to the electronic supplementary material.

Supplementary file1 (DOCX 19 KB)

Supplementary file2 (DOCX 36 KB)

## Data Availability

Ethical approval was not received for sharing of the data for this study.
