# Supplementary material for: Acoustic and Perceptual Profiles of Swallowing Sounds in Preterm Neonates: A Cross-Sectional Study Cohort
Source: Dysphagia. 2025 Feb 11;40(5):1113–23. doi: 10.1007/s00455-025-10807-5 (PMC12479640; doi:10.1007/s00455-025-10807-5)
Supplement: Supplementary file 1 — Supplementary file1 (DOCX 19 KB) [file 455_2025_10807_MOESM1_ESM.docx]

**Supplemental Table A:** Inter-rater reliability for two raters

|  | **Perceptual sound parameter** | **N^a^** | **Agreement (%)** | **Cohen’s kappa**  **K, (95% CI)** | **PABAK^b^**  **Coefficient, (95% CI)** |
| --- | --- | --- | --- | --- | --- |
| Pre-swallow | Normal breathing | 80 | 77.50 (68.15, 86.85) | 0.45, (0.23, 0.67) | 0.55, (0.36, 0.74) |
|  | Wet breathing | 78 | 93.59 (88.03, 99.15) | 0.42, (0.01, 0.83) | 0.87, (0.76, 0.98) |
|  | Rattly chest | 80 | 100 | Not estimable | Not estimable |
|  | Grunting | 79 | 91.03 (84.73, 97.55) | <0 | 0.82, (0.69, 0.95) |
|  | Crackles | 80 | 100 | Not estimable | Not estimable |
|  | Stridor | 79 | 84.81 (76.62, 92.90) | 0.51, (0.26, 0.75) | 0.70, (0.53, 0.86) |
|  | Wheeze | 80 | 87.50 (80.09, 94.91) | <0 | 0.75, (0.60, 0.90) |
|  | Coughing | 80 | 100 | Not estimable | Not estimable |
|  | Throat clearing | 80 | 100 | Not estimable | Not estimable |
|  |  |  |  |  |  |
| During swallowing | Crisp & clear distinct | 80 | 85.00 (77.00, 93.00) | 0.25, (-0.06, 0.56) | 0.70, (0.54, 0.86) |
|  | Quick | 78 | 100 | Not estimable | Not estimable |
|  | Load | 80 | 81.25 (72.51, 89.99) | 0.38, (0.12, 0.63) | 0.63, (0.45, 0.80) |
|  | Initial discrete sound | 80 | 97.50 (94.00, 100.00) | 0.33, (0.02, 0.64) | 0.96, (0.91, 1.00) |
|  | Bous transit sound | 79 | 100 | Not estimable | Not estimable |
|  | Final discrete sound | 79 | 100 | Not estimable | Not estimable |
|  | Glottal release sound | 78 | 97.44 (93.85, 100.00) | 0 (0, 0) | 0.95, (0.88, 1.00) |
|  | Co-ordinated | 77 | 83.12 (74.56, 91.68) | 0.42, (0.15, 0.69) | 0.66, (0.49, 0.83) |
|  | Unco-ordinated | 77 | 83.12 (74.56, 91.68) | 0.42, (0.15, 0.69) | 0.66, (0.49, 0.83) |
|  |  |  |  |  |  |
| Post-swallow | Normal breathing | 80 | 86.25 (78.54, 93.96) | 0.72, (0.56, 0.88) | 0.72, (0.56, 0.88) |
|  | Wet breathing | 79 | 92.41 (86.43, 98.38) | 0.74, (0.54, 0.94) | 0.85, (0.73, 0.97) |
|  | Rattly chest | 80 | 100 | Not estimable | Not estimable |
|  | Grunting | 78 | 85.90 (78.00, 93.80) | <0 | 0.72, (0.56, 0.88) |
|  | Crackles | 80 | 100 | Not estimable | Not estimable |
|  | Stridor | 79 | 84.81 (76.72, 92.90) | 0.61, (0.41, 0.81) | 0.70, (0.53, 0.86) |
|  | Wheeze | 80 | 83.75 (75.49, 92.01) | <0 | 0.68, (0.51, 0.84) |
|  | Coughing | 80 | 96.25 (92.00, 100.00) | 0.65, (0.28, 1.00) | 0.93, (0.84, 1.00) |
|  | Throat clearing | 80 | 98.75 (96.26, 100.00) | 0, (0, 0) | 0.98, (0.93, 1.00) |

^a^ A complete-case analysis approach was undertaken, meaning that a participant was excluded from the analysis if one or more raters could not assess the participant as a yes or no.

^b^ Prevalence-adjusted bias-adjusted kappa agreement coefficient. It is also called the Brennan and Prediger agreement coefficient.
