# Supplementary material for: Acoustic and Perceptual Profiles of Swallowing Sounds in Preterm Neonates: A Cross-Sectional Study Cohort
Source: Dysphagia. 2025 Feb 11;40(5):1113–23. doi: 10.1007/s00455-025-10807-5 (PMC12479640; doi:10.1007/s00455-025-10807-5)
Supplement: Supplementary file 2 — Supplementary file2 (DOCX 36 KB) [file 455_2025_10807_MOESM2_ESM.docx]

Supplemental Table B: Pre-swallow respiratory, swallow and post-swallow respiratory sounds definitions

| Pre- and post-swallow sounds | Medical definition | Lay definition |
| --- | --- | --- |
| Normal breathing | Normal, good, unlabored [respiratory](http://en.wikipedia.org/wiki/Breath) pattern, sometimes known as *quiet breathing* or *resting respiration*^1^ | When the inspiration and expiration sequence sounds clear^2^ |
| Wet breathing | Breathing has a wet sounding quality | Breathing sounds wet |
| Rattly chest | Bronchial fremitus is a palpable vibration produced during breathing, caused by partial airway obstruction^3^ | Vibration on palpation when hands are placed on the infant’s chest/back |
| Grunting | A repetitive, low to medium pitch, short, explosive sound which is produced by vocal cord closure during expiration^4^ | Vocalization heard with expiratory breath^5^ |
| Crackles - fine | Inspiratory crepitations^5^ | Similar to sound of wood burning in a fireplace or the sound of cellophane being crumpled^6^ |
| Crackles - coarse | Low in pitch, bubbling sound^5^ | Similar to sound when strands of hair are rolled between your fingers near your ear or similar to the sound of velcro being pulled apart^6^ |
| Stridor | Harsh, monophonic, predominantly inspiratory noise. Can be expiratory or biphasic^5^ | A harsh, high-pitched, vibratory noise in the throat, particularly when breathing in. Can be when breathing in or out or both^7^ |
| Wheeze | Continuous, high-pitched adventitious lung sounds which are superimposed on the normal breath sounds^8^ | Flow of air through an airway sounds turbulent^5^ |
| Coughing | A cough that sounds barking/brassy, honking, paroxysmal, staccato or wet^9^ | Expelling air from the lungs suddenly with a harsh noise |
| Throat clearing | Throat clearing | An acute expelling of sound from the throat |

| During swallow sounds | Description | Listening tips |
| --- | --- | --- |
| Crisp & clear ‘distinct’ | Sounds clear^10^ |  |
| Quick | Swallow occurs within 1 second^11^ |  |
| Loud | Sound is strongly audible | May vary depending on whether the texture is a fluid, puree or solid |
| Initial discrete sound (IDS) | Click^12^ | Soft, short sound similar to initial sound heard when ears are ‘unblocked’ |
| Bolus transit sound (BTS) | Food or fluid flushing sound^12, 13^ *or*  Flow sound related to turbulences of the fluid^14^ | May vary depending on whether the texture is a fluid, puree or solid - will be more audible on a fluid bolus |
| Final discrete sound (FDS) | Click^12^ | Soft, short sound similar to initial sound heard when ears are ‘unblocked’ |
| Glottal release (GRS) | Short expiration burst of air^10^ | May vary depending on whether the child is taking a single swallow or multiple swallows. Should be more identifiable on single swallows |
| Co-ordinated | Presence of a BTS and normal breathing post-swallow or no change to breath sounds pre- and post-swallow.^13^ |  |
| Unco-ordinated | Presence of a BTS and an absence of normal breathing post-swallow and/or GRS plus one or more of the following post-swallow: wet breathing, rattly chest, cough, wheeze, crackles, throat clearing, stridor.^13^ |  |

1. In: Lukens R, (ed.). *Stedman's Medical Dictionary* 28 ed. Maryland: Lippincott Williams & Wilkins, 2006, p. 677.

2. Rommel N. *Diagnosis of oropharyngeal disorders in young children*. Doctor of Philosophy, Katholieke Universiteit Leuven, Kindergeneeskunde, 2002.

3. *Dorland's Illustrated Medical Dictionary*. 32 ed.

4. Poole SR, Chetham M and Anderson M. Grunting respirations in infants and children. *Pediatr Emerg Care* 1995; 11: 158-161.

5. O'Callaghan C and Stannard W. Paediatric Respiratory Examination. BMJ books, 2001.

6. Easy Auscultation, <http://www.easyauscultation.com> (2010, accessed 16.01.2012).

7. Weir K. Children's Feeding Skills Questionaire. Brisbane 2005.

8. Meslier N, Charbonneau G and Racineux J-L. Wheezes. *Eur Respir J* 1995; 8: 1942-1948.

9. Chang AB, Landau L, Asperen P, et al. Cough in children: definitions and clinical evaluation. Position statement for the Thoracic Society of Australia and New Zealand. *Med J Aust* 2006; 184: 398-403.

10. Cichero JAY and Murdoch BE. Acoustic signature of the normal swallow: characterization by age, gender, and bolus volume. *Ann Otol Rhinol Laryngol* 2002; 111: 623-632.

11. Frakking TT, Chang AB, O'Grady KF, et al. Acoustic and Perceptual Profiles of Swallowing Sounds in Children: Normative Data for 4-36 Months from a Cross-Sectional Study Cohort. *Dysphagia* 2017; 32: 261-270. 2016/11/11. DOI: 10.1007/s00455-016-9755-1.

12. Vice FL, Heinz JM, Giuriati G, et al. Cervical auscultation of suckle feeding in newborn infants. *Dev Med Child Neurol* 1990; 32: 760-768.

13. Frakking T, Chang A, O'Grady K, et al. Aspirating and Nonaspirating Swallow Sounds in Children: A Pilot Study. *Ann Otol Rhinol Laryngol* 2016; 125: 1001-1009. 2016/09/30. DOI: 10.1177/0003489416669953.

14. Moriniere S, Boiron M, Alison D, et al. Origin of the sound components during pharyngeal swallowing in normal subjects. *Dysphagia* 2008; 23: 267-273. Research Support, Non-U.S. Gov't.
